# Co-existence of physical activity and sedentary behavior among children and adolescents in Shanghai, China: do gender and age matter?

**DOI:** 10.1186/s12889-018-6167-1

**Published:** 2018-11-22

**Authors:** Si-Tong Chen, Yang Liu, Jin-Tao Hong, Yan Tang, Zhen-Bo Cao, Jie Zhuang, Zheng Zhu, Pei-Jie Chen

**Affiliations:** 1 School of Physical Education and Sport Training, Shanghai University of Sport, Shanghai, 200438 China; 2 Shanghai Research Centre for Physical Fitness and Health of Children and Adolescents, Shanghai University of Sport, Shanghai, 200438 China; 3 School of Kinesiology, Shanghai University of Sport, Shanghai, 200438 China

**Keywords:** Moderate-to-vigorous physical activity (MVPA), Screen time (ST), Clusters of behavior, Factors, School-aged children and adolescents, Shanghai

## Abstract

**Background:**

There is limited evidence for the prevalence of the co-existence of physical activity (PA) and sedentary behavior (SED), and its correlates among children and adolescents. This study has two aims: 1) to investigate the prevalence of PA and SED, and their co-existence, and 2) to examine the associations between PA or SED, or both with gender and age among children and adolescents in Shanghai, China.

**Methods:**

Using a cross-sectional study design (conducted from September to December 2014), 50,090 children and adolescents (10–18 years old, 50.4% boys) were included in this study. A self-reporting questionnaire was used to measure participants’ sociodemographic characteristics, PA, and SED. Descriptive statistics were used to describe sample characteristics, the prevalence of PA and SED, and their co-existence. A Generalized Linear Model was conducted to explore the associations between the prevalence of PA and SED, and their co-existence with gender and age separately.

**Results:**

Of the children and adolescents studied, only 18.4% met the guidelines for PA, 25.5% met the guidelines for SED, and 5.7% met the guidelines for both. Boys were more physically active (aOR = 1.43, 95% CI: 1.36–1.50), and girls were less sedentary (aOR = 1.29, 95%CI: 1.24–1.34). The prevalence of PA, SED, or both all declined as age increased (*p* < 0.001). Stratified analysis by gender revealed greater declining trends of meeting the PA or SED guidelines, or both in girls (all *p* < 0.005).

**Conclusion:**

Very few children and adolescents showed active lifestyles, and this was significantly related to age. Effective interventions aiming to promote PA and concurrently to limited SED among children and adolescents should be implemented as early as possible.

## Background

Physical activity (PA) is a complex behavior that includes sedentary behavior (SED) [1]. Children and adolescents gain diverse health benefits from sufficient PA [2] and limited SED [3]. PA and SED can be tracked from childhood into adulthood [4]. The World Health Organization (WHO) recommends that people from 5-to-17 years old should accumulate at least 60 min daily of moderate-to-vigorous physical activity (MVPA) [5], and the Canadian 24-Hour Movement guidelines [6] suggest that daily screen time (ST) should be limited to below 2 h per day. Despite established evidence and guidelines, a significant decrease in PA [7] and an increase in SED [8] have been observed over time. Recent global studies showed poor levels of PA and SED [9, 10], and parallel circumstances emerged out in some developed countries and regions as well [11–13].

Low levels of PA have been marked among children and adolescents in Asia. For example, one Japanese study using accelerometers found that children spent over 50% of wear-time on SED per day, whereas the percentage of MVPA time was 7.9% [14], which was similar to one Korean study [15]. China, the biggest country in Asia, has suffered the burden of young people’s insufficient PA and excessive SED over the past decades [16], which has aroused the concerns of policy-makers [17, 18]. To our knowledge, Tudor-Lock et al. [16] first reported the pattern of PA in Chinese school-aged children and adolescents internationally. Since that, interest in examining the prevalence of PA and SED among Chinese young people have increased. One study showed decreasing PA time and increasing SED time among Chinese young people from 2006 to 2010 [19]. Two Chinese national studies reported that 29.9% of children and adolescents met the PA guidelines [20] and 63.2% met the SED guidelines [21]. These poor results were similar to the results from two regional studies [22, 23].

Researchers have called for effective interventions to battle against inactive behavior among young people [17, 24]. Prior to effective PA and SED interventions, correlates of PA and SED must be fully understood [17, 25]. The established Social Ecological Model (SEM) provides a theoretical framework to interpret correlates of PA [26]. Some studies identified the correlates of PA and SED among Chinese children and adolescents [19–21, 23, 27], but some limitations of these studies remain [27]. Also, one systematic review indicated that subjectively measured SED was not associated with gender [28], which was inconsistent with some recent Chinese studies [19, 20, 23]. As mentioned by Verloigne et al. [29], there were great variations in SED among children and adolescents from different European countries (regions), implying cultural and regional factors of affecting SED [30]. In this line, more specific interpretations regarding the correlates of SED among different populations from different regions are needed. Additionally, the previous studies were not conducted in Shanghai where are high levels of urbanization and industrialization. This lack of evidence inhibits the understanding of correlates of SED among children and adolescents in Shanghai.

Increasing PA and decreasing SED among children and adolescents have been simultaneously dual priorities [31], which have aided in identifying the sub-populations who are threatened by health risks [32]. Hence, the co-existence of different behaviors (e.g., PA, SED, and diet) has gained much attention [33], because those behaviours are significantly associated with health outcomes [34–36]. However, little is known about the co-existence of PA and SED, and their potential correlates among Chinese children and adolescents. So far, only the ISCOLE international study (including China) determined that 16.6% of children aged 9–11 years old met both the PA and SED guidelines, which was higher than the Chinese level (6.2%) [34]. Nevertheless, the age range of samples in the study was relatively small, so we could not identify the sub-populations. As simultaneously meeting the PA and SED guidelines has public health implications [33], such as reducing the odds of being obese [34], the examinations of co-existence support an understanding of healthy lifestyles and how to design effective strategies for such lifestyles.

To investigate PA and SED for designing and implementing effective interventions for young Chinese people, therefore, the purposes of this study are: 1) to update the prevalence of PA and SED and their co-existence; and 2) to examine the association between PA and SED, and their co-existence with gender and age groups (because of their popularity across previous studies).

## Methods

### Study design and participants

This study was a large cross-sectional school survey conducted from September to December 2014 in Shanghai, China in which 1st-12th graders in 711 public primary, middle, and high schools were selected from all 17 districts in Shanghai, using a multistage stratified and random cluster sampling method. Since this study was organized by the Shanghai Municipal Education Commission, no school declined to participate. Of all the students, 78,516 students (aged 6 to 18 years) were invited to participate in the survey. In response, 71,404 students (response rate = 90.9%) completed the self-reported questionnaire. The reasons given by the remaining students for not completing the questionnaire were: 1) some participants were taking academic tests when we were collecting data, so those students were unable to take part in the survey; and 2) some participants did not re-submit the questionnaire due to absenteeism caused by physical diseases. The detailed information concerning the study design can be found elsewhere [22]. The study protocol and procedure were approved by the Institutional Review Board (IRB) of the Shanghai University of Sport (SUS), and permission to conduct the study was obtained from the teachers and principals of the participating schools. The IRB of SUS approved that the verbal consent is sufficient to conduct this study due to the fact that none of survey items related to personal ethic issue. All the children involved in the study, and their parents or guardians, were specifically advised that participation was completely voluntary. Verbal informed consent was obtained from all parents or guardians, and positive assent was obtained verbally from all the children prior to data collection. Data were collected and analysed anonymously.

### Measurement of variables

To measure PA and SED, this study employed the related-items derived from the Health Behavior School-aged Children (HBSC) survey questionnaire. Those items have been confirmed as feasible and reliable measures of PA and SED for Chinese children and adolescents [37]. Participants were required to report their information on gender (responses: 1 = male, 2 = female), grade (responses: 1, 2, 3, … 12). *The questionnaire used the following two items to collect information on PA: 1) How many days did you engage in MVPA at least 60 min on weekdays over the past week? (responses: 0 = none, 1 = 1 day, 2 = 2 days, 3 = 3 days, 4 = 4 days, 5 = 5 days); 2) How many days did you engage in MVPA at least 60 min on the weekend over the past week? (responses: 0 = none, 1 = 1 day, 2 = 2 days).* To help participants better understand MVPA, it was explained as “*any kind of physical activity that increased your heart rate and made you breathe hard some of the time (including physical education time, exercising, sports training, and various regular daily activities such as brisk walking, hiking, and excursion)*.” Consistent with the WHO PA recommendation [5] and the Canadian 24-Hour Movement Guideline [6], the definition of meeting the MVPA recommendation was that participants reported 7 days with 60 min of MVPA daily. The prevalence of PA was defined as the percentage of the participants who met MVPA recommendations in the total sample population.

The questionnaire used the following items to collect information on SED: *1) How many hours did you spend in watching TV or movies in your leisure time on weekdays and on the weekend over the past week? (responses: 1 = none, 2 = about half an hour, 3 = about one hour, 4 = about two hours, 5 = about three and more hours); 2) How many hours do you spend in playing computer games in your leisure time on weekdays and on the weekend over the past week? (responses: same as the above item); 3) How many hours do you spend in activities using electronic screen-based devices in leisure time on weekdays and on the weekend over the past week? (responses: same as the above item).* Consistent with the Canadian 24-Hour Movement Guidelines [6], the definition of meeting the SED guidelines was that the daily screen time of participants, including time watching TV/movies, playing computer games and using electronic screen-based devices, was less than 2 h per day. The prevalence of SED was defined as the percentage of participants who met the SED guidelines in the sample population.

Consistent with Canadian 24-Hour Movement Guideline [6], the definition for meeting the PA and SED recommendations was that individuals simultaneously have 60 min (or more) of MVPA daily and less than 2 h of screen time per day.

### Statistical analysis

All of the statistical analyses were conducted in SPSS (Chicago, IL, USA). According to the aims of this study, variables of age (grade), gender, PA, and SED were included in the statistical analysis. Two phases of data filtering were conducted. Phase 1 was to remove the missing cases and abnormal values: deleting missing cases (or having abnormal values) for grade (*n* = 280), gender (*n* = 255), PA on weekdays and weekend days (*n* = 1458), time watching TV/movies on weekdays and weekend days (*n* = 2322), time playing computer games on weekdays and weekend days (*n* = 503), and time using electronic screen-based activities on weekdays and weekend days (*n* = 293). Phase 2 was to exclude the younger participants because of measurement validity. Considering the low validity of these measurements for children under 10 years old (1st-3rd graders), those participants (*n* = 16,784) were excluded. In total, 50,090 eligible cases were included in the analytical dataset. Participants were categorized into 3 age groups (younger adolescents: grades 4–5; adolescents: grades 6–9; older adolescents: grades 10–12) to reflect the levels in Shanghai, China. There were no differences in age or gender between those who completed the questionnaire and those who did not.

In accordance with the Canadian 24-Hour Movement Guidelines [6], the co-existence of PA and SED was classified into three clusters. Cluster 1: meet both the PA and SED guidelines (coded as 1; definition: daily MVPA time was ≥60 min while daily screen time was < 2 h); cluster 2: meet either PA or SED guidelines (coded as 2; definition: daily MVPA time was ≥60 min while daily screen time was ≥2 h, or daily MVPA time was < 60 min while daily screen time was < 2 h); and cluster 3: meet neither PA nor SED guideline (coded as 3, definition: daily MVPA time < 60 min while daily screen time was ≥2 h). Descriptive statistics were used to describe sample characteristics, the prevalence of PA, SED, and clusters. A chi-square test was applied to examine the gender and age difference in the prevalence of PA and SED, and their co-existence. A Generalized Linear Model was performed to examine the association of PA and SED, and their co-existence with gender and age. Statistical significance was set at *p* < 0.05.

## Results

The characteristics of the sample (*n* = 50,090) in this study are shown in Table 1. The percentage of boys and girls in the total samples were 50.4 and 49.6%, respectively. The highest proportion of different age groups was found in adolescent group (43.7%), as well as boys (43.9%) and girls (43.4%). In the younger adolescent and adolescent groups, there were more boys than girls (29.7% vs 28.4%; 43.9% vs 43.4%, respectively). There was a statistically significant gender difference among age groups (*p* < 0.001).

**Table 1:** The Characteristics of the Samples

	Total		Boys		Girls	
	n	%	n	%	n	%
Total	50,090	100	25,244	50.4	24,846	49.6
Age Groups^***^						
Younger adolescents	14,552	29.1%	7486	29.7%	7066	28.4%
Adolescents	21,879	43.7%	11,088	43.9%	10,791	43.4%
Older adolescents	13,659	27.3%	6670	26.4%	6989	28.1%

Younger adolescents: 4-5th graders

Adolescents: 6-9th graders

Older adolescents: 10-12th graders

Significance difference at genders at *p* < 0.001

*p < 0.05, **p < 0.01, ***p < 0.001

Table 2 presents the prevalence of PA and SED by gender and age groups. 18.4 and 25.5% of children and adolescents met the PA and SED guidelines, respectively. The percentage of boys meeting the PA guidelines was higher than girls (21.0% vs 15.7%, *p* < 0.001), but lower in the SED guidelines (23.3% vs 27.8%; *p* < 0.001). The percentages of meeting the PA guidelines in the three age groups were different (younger adolescents: 33.0%; adolescents: 16.2%; older adolescents: 6.5%). The percentage of meeting the SED guidelines in the three age groups were different (younger adolescents: 35.4%; adolescents: 22.0%; older adolescents: 19.6%). There was a statistically significant age difference in the prevalence of PA and SED (*p* < 0.001).

**Table 2:** The Prevalence of Meeting the PA and SED Guidelines

	PA^a, b^		SED^a, b^	
	n	%	n	%
Total	9216	18.4	12,772	25.5
Gender				
Boys	5309	21.0	5874	23.3
Girls	3907	15.7	6898	27.8
Age Groups				
Younger adolescents	4796	33.0	5154	35.4
Adolescents	3538	16.2	4935	22.6
Older adolescents	882	6.5	2683	19.6

Younger- adolescents: 4-5th graders

Adolescents: 6-9th graders

Older- adolescents: 10-12th graders

^a^denotes significant gender difference at *p* < 0.001

^b^denotes significant age group difference at *p* < 0.001

The prevalence of the three clusters is shown in Table 3. The prevalence of meeting the clusters 1, 2 and 3 were 5.7, 32.4, and 61.8%, respectively. The proportion of boys who were in cluster 1 was only 5.7%, which was equal to that in girls (5.7%). The percentages of cluster 2 in boys and girls were 32.8 and 32.0%, respectively. The percentage of cluster 3 in girls was 62.2%, which was slightly more than that in boys (61.4%). There was no statistically significant gender difference across different clusters (*p* = 0.14). The percentages of cluster 1 among the three age groups were 13.2, 3.5, and 1.4%, respectively. Regarding cluster 2, the percentages of the three age groups decreased (younger adolescents: 42.0%; adolescents: 31.8%; older adolescents: 23.2%). In cluster 3, the percentages of the three age groups increased with age (younger adolescents: 44.8%; adolescents: 64.7%; older adolescents: 75.3%; *p* < 0.001).

**Table 3:** The Prevalence of Clusters by Gender, Age Groups

	Cluster 1		Cluster 2		Cluster 3	
	n	%	n	%	n	%
Total	2872	5.7	16,244	32.4	30,974	61.8
Gender						
Boys	1447	5.7	8289	32.8	15,508	61.4
Girls	1425	5.7	7955	32.0	15,466	62.2
Age Groups^***^						
Younger adolescents	1917	13.2	6116	42.0	6519	44.8
Adolescents	758	3.5	6957	31.8	14,164	64.7
Older adolescents	197	1.4	3171	23.2	10,291	75.3

Younger adolescents: 4-5th graders

Adolescents: 6-9th graders

Older adolescents: 10-12th graders

Cluster 1: meet both the PA and SED guidelines, signifying that daily MVPA time was ≥60 min while daily screen time was < 2 h

Cluster 2: meet either PA or SED guidelines, signifying that daily MVPA time was ≥60 min while daily screen time was ≥2 h, or daily MVPA time was < 60 min while daily screen time was < 2 h

Cluster 3: meet neither PA nor SED guideline, signifying that daily MVPA time < 60 min while daily screen time was ≥2 h

Significant age group difference across the clusters at *p* < 0.001

*p < 0.05, **p < 0.01, ***p < 0.001

Generalized linear model analyses revealed the associations between PA and SED, and their co-existence with gender and age (Table 4). Compared to girls, boys had a 43% (OR = 1.43, 95% CI: 1.36–1.49) better chance of meeting the PA guidelines. Children and adolescents in the younger adolescent and adolescent groups were 7.12 and 2.79 times greater in meeting the PA guidelines than those in the older adolescent group. For SED, girls were 1.27 (OR = 1.12, 95% CI: 1.07–1.17) times more likely than boys to meet the SED guidelines compared to boys. Among children and adolescents who were in the younger adolescent (OR = 2.24, 95% CI: 2.13–2.37) and adolescent groups (OR = 1.19, 95% CI: 1.13–1.26), both had a greater likelihood of meeting the SED guidelines. However, in comparison to the older adolescent group, children who were in the younger adolescent (OR = 4.11, 95% CI: 3.91–4.32) and adolescent groups (OR = 1.67, 95% CI: 1.59–1.75) were significantly more likely to meet both the PA and SED guidelines or meet either of the two guidelines compared to the adjacently worse cluster.

**Table 4:** Associations of Gender, Age groups with the Prevalence of meeting PA, SED, and Their Co-existence of Recommendation Guidelines

	PA						SED						Co-existence					
	uOR	95%CI		aOR	95%CI		uOR	95%CI		aOR	95%CI		uOR	95%CI		aOR	95%CI	
Gender																		
Boys	1.43	1.36	1.49	1.43	1.36	1.5	1			1			1.03	1	1.07	1.01	0.97	1.05
Girls	1			1			1.27	1.22	1.32	1.29	1.24	1.34	1			1		
Age Groups																		
Younger adolescents	7.12	6.6	7.69	7.12	6.59	7.68	2.24	2.13	2.37	2.26	2.15	2.39	4.11	3.91	4.32	4.11	3.91	4.32
Adolescents	2.79	2.59	3.02	2.79	2.58	3.01	1.19	1.13	1.26	1.2	1.14	1.26	1.67	1.59	1.75	1.67	1.59	1.75
Older adolescents	1						1						1					

Younger adolescents: 4-5th graders

Adolescents: 6-9th graders

Older adolescents: 10-12th graders

uOR: unadjusted odds ratio

aOR: adjusted odds ratio (age and gender were controlled respectively)

CI: confidence intervals

Reference category: Gender: Girl (except for SED), age group: Older adolescents

The results of the generalized linear models by gender are presented in Table 5. Boys in the younger adolescent and adolescent groups were about 4.77 (95% CI: 4.33–5.24) and 2.33 (CI: 2.12–2.56) times more likely to meet the PA guidelines than those in the older adolescent group, respectively. Also, younger girls were more likely to meet the PA guidelines, especially those in the younger adolescent group, who were 13.11 times more likely (OR = 13.11, 95% CI: 11.44–15.02) than those in the older adolescent group and 9.19 times greater than the adolescent group. In terms of the odds for meeting the SED guidelines, boys in the younger adolescent and adolescent groups were 1.97 times (OR = 1.97, 95% CI: 1.82–2.13) and 1.12 times more likely than those in the older adolescent group. Among girls, the odds of meeting the SED guidelines among the younger adolescent and adolescent groups were 2.56 (95% CI: 2.38–2.76) and 1.26 (95% CI: 1.18–1.36) times greater, respectively, than for the older adolescent group. Across different clusters, generalized linear models showed that boys (OR = 1.61, 95% CI: 1.51–1.72) and girls (OR = 1.72, 95% CI: 1.61–1.84) in the adolescent group displayed similar trends in having better clusters of behaviors. By contrast, boys in the younger adolescent group were more likely to show better clusters of behaviors (OR = 3.38, 95% CI: 3.15–3.62), but this value was larger for girls (OR = 5.04, 95% CI: 4.69–5.42).

**Table 5:** Associations of Age Groups with the Prevalence of Meeting PA and SED, and Their Co-existence Recommendation Guidelines by Gender

	PA			SED			Co-existence		
	OR	95% CI		OR	95% CI		OR	95% CI	
Boys									
Younger adolescents	4.77	4.33	5.24	1.97	1.82	2.13	3.38	3.15	3.62
Adolescents	2.33	2.12	2.56	1.12	1.04	1.21	1.61	1.51	1.72
Older adolescents	1			1			1		
Girls									
Younger adolescents	13.11	11.44	15.02	2.56	2.38	2.76	5.04	4.69	5.42
Adolescents	3.92	3.41	4.50	1.26	1.18	1.36	1.72	1.61	1.84
Older adolescents	1			1			1		

Younger adolescents: 4-5th graders

Adolescents: 6-9th graders

Older adolescents: 10-12th graders

OR: odds ratio

CI: confidence intervals

Reference category: Older adolescents

## Discussion

This study examined the co-existence of PA and SED among a large-sized sample of children and adolescents in Shanghai, China. Our results showed that very few children and adolescents in Shanghai met both PA and SED guidelines, and that age was negatively associated with the prevalence of meeting the PA and SED guidelines, and their co-existence.

To our knowledge, this is the first study to investigate the prevalence of PA and SED, and their co-existence among children and adolescents in China. Overall, only a few children and adolescents met generally acceptable PA and SED guidelines, both of which were lower than the published international trends [9, 10] as well as Chinese levels [20, 21]. When we combined PA and SED, very few children and adolescents met both PA and SED guidelines, but alarmingly, the majority of children and adolescents met neither PA nor SED guidelines. This finding suggests poor PA behaviors among Chinese children and adolescents. Prior research investigating the co-existence of PA and SED used cluster analyses (CA) and latent cluster analyses (LCA) [33], which provide little directly comparable evidence for our study deriving from PA and SED guidelines. So far, only one study applying the PA and SED guidelines to determine international and Chinese co-existence showed better levels of PA and SED than our study [34]. The poor levels of PA and SED among children and adolescents in Shanghai might be explained by rapid urbanization, economic growth and societal transformation [38]. It is strongly recommended that more children and adolescents should meet both the PA and SED guidelines [39]. Effective interventions should be promoted among children and adolescents in China. Most current studies have not combined PA and SED in their analyses due to their complex relationships [37]. Since concurrently meeting both PA and SED guidelines has advantages over meeting one or neither of the guidelines—such as decreasing the odds of being obese [34, 40] and improving aerobic fitness [40]—only the promotion of PA or decrease in SED might generate limited health benefits. Since a conceptual framework aggregating PA and SED was established [1], future research focusing on the co-existence of PA and SED is now both possible and needed [33]. The development of a multi-dimensional intervention to promote PA behavior in Chinese children and adolescents is needed to promote healthy lifestyles.

Consistent with other studies [20, 22, 23], we found that boys were more active than girls, which might be explained by greater participation in active transportation [31], organized sports activities [22], and a higher level of MVPA in physical education lessons [41]. The finding that boys were more sedentary than girls in this study was inconsistent with other studies [23, 31, 42] and might be explained by measures that lack questions specific to activities that girls’ enjoy to potentially reduce their actual SED time [43]. Consequently, extensive measures reflecting girls’ daily SED are needed in future studies. There was no significant gender difference in co-existence in this study, which is inconsistent with previous studies [32, 35]. This disparity might be attributable to different measures and statistical techniques. For example, Patnode et al. [35] used accelerometers to measure participants’ PA and determined clusters by LCA, generating different clusters. Despite the fact that most current studies regarding the co-existence of PA and SED used similar statistical techniques, methodological limitations in statistics still exist [33]. It is suggested that future studies determining clusters of behavior should adopt objective measures and the PA and SED guidelines for children and adolescents. Due to the low prevalence of meeting the combinations of PA and SED, this study demonstrates that unhealthy lifestyles were highly prevalent regardless of gender. We also observed an interesting finding for a similar prevalence by gender in cluster 1 and cluster 3, which might have been caused by different distributions of PA time characterized by gender. In other words, gender was not the correlate of the co-existence of PA and SED. Hence, future studies should further examine how Chinese boys and girls allot their time to PA behavior as well as the relationship between gender and the co-existence of PA and SED. In light of these findings, our study suggests that interventions aiming to promote active lifestyles should pay equal attention to boys and girls without gender-related priority. Meanwhile, gender-specific strategies should be considered when promoting PA or limiting SED.

Age was not a convincing factor related to PA among children and adolescents; however, it was difficult to draw a consensus conclusion due to the limited age range in previous studies [27]. One study indicated that PA among children over 5 years old declined with age [8]. Considering our samples’ age (10—18 years old), we can confirm that PA decreased as age increased, which was caused by growing academic pressure [16, 27]. Also, the popular use of screen devices for entertainment, communication, and homework among older adolescents in Shanghai could also increase SED. The findings of this study indicate that age is related to the co-existence of PA and SED. Significant age differences across different clusters were found, which is consistent with previous studies [32, 35]. Since an age-related decrease in the prevalence of PA and SED in this study conjointly resulted in reduced prevalence in cluster 1, we can confirm that age is a negative factor related to the co-existence of PA and SED. Very few older adolescents met both the PA and SED guidelines, while the majority met only one of the two guidelines. Together, this implies severely unhealthy lifestyles among older adolescents. In this regard, high priority should be given to changing behavior among older adolescents. In addition, this study found that a more evident decrease in the prevalence of cluster 1 and an increase in the prevalence of cluster 3 in the early transition (from younger adolescents to adolescents) than the later transition (from adolescents to older adolescents). This denotes greater declining trends of meeting better cluster during the early transition. To better develop an active lifestyle, effective interventions in lifestyle should be initiated before adolescence to develop early PA habits [44]. Two studies revealed a lower stability of PA in childhood and early adolescence than in the later period [45, 46]. Increasing academic pressure and reduced opportunities for PA participation are potential reasons for this trend. In Shanghai, children and adolescents face much heavier academic tasks after the younger-adolescents period, which reduces opportunities for PA and augments SED time. Despite the proposed theoretical factors relating to changes in PA and SED [45–47], uncertainty remains regarding their determinants [7]. More robust evidence of changes in PA and SED among Chinese children and adolescents in Shanghai is needed. Such knowledge can be beneficial for explaining the different trends over different periods.

Relative to boys, the declining trends in meeting separate PA and SED guidelines as well as combined guidelines were larger in girls in early transition. Gender differences in the tracking of PA were discovered [7]. The extent of diligence in studying was an important consideration. Among adolescents and older-adolescents, girls spent more time than boys in studying, which occupied their time for PA. Sallis [48] also stated that the pubertal period with sexual maturity in girls occurs earlier than in boys, which accounted for the declining trend among girls [49]. However, it is not clear yet which factors are related to changes in girls [7]. Due to the different factors of PA in boys and girls [50, 51], a more comprehensive investigation of the significant PA decline in girls is needed. Similarly, the extent of declining trends in SED among girls was more striking than for boys. In light of knowledge gaps concerning SED [28], uncertainty about the more rapid increase in SED among girls remains. The behavior preferences of girls might be responsible. It should be noted that preventing the dramatic declines in the PA in older girls is a favourable approach for public health concerns. Therefore, the transition from younger-adolescence to adolescence is a critical time for implementing interventions [44].

We used a self-reported questionnaire to assess PA and SED. Instruments to measure PA and SED are classified as objective and subjective [51–55]. Objective measures (e.g., accelerometers) overcome the limitations of subjective measures (e.g., self-reported questionnaires), especially in overestimating PA level and underestimating SED level [52–54], which have resulted in discrepancies among various studies. However, the distinctive merits of self-reported measures, including the low cost and convenience of administration [52, 54], have made studies with large samples more feasible [56]. To date, the majority of previous Chinese studies used self-reported questionnaires to measure PA and SED [16, 20, 22, 23]. Thus, it is reasonable that the current study used self-reported questionnaires for measurement. Future studies should use objective measures to estimate PA and SED. Additionally, concerns about measurement issues should be considered when comparing findings among studies using different measures.

The first strength of this study was the large size of representative samples covering a broad range of age, which reflected trends in the prevalence of PA behavior across different age groups. Moreover, to our knowledge, this is one of the few studies to examine the co-existence of PA and SED among Chinese young people. Finally, we investigated changes in prevalence to meet the single and combined guidelines to inspect the risk of this sub-population needing prioritized interventions. Despite these strengths, some limitations should be mentioned. First, this study only represented Shanghai, so its generalization is limited to this region [22]. Additionally, the prevalence of PA and SED was estimated by a self-reported questionnaire, which may influence the accuracy of estimation. Furthermore, due to limitations in the questionnaire used in this study, we were not able to determine more information regarding demographic variables, such as body mass index (BMI), socio-economic factors, types of schools and urban/rural gradients. Finally, this study did not establish a causal relationship due to the cross-sectional study design. Therefore, further research should adopt improved study designs that include objective measures, longitudinal observation, more independent variables and samples expanded to various regions in China.

## Conclusion

This study showed that the majority of children and adolescents in Shanghai were both physically inactive and sedentary. Gender was an important correlate of PA and SED. Age was negatively associated with co-existence of PA and SED. Declining trends in the prevalence of PA and SED as well as their co-existence consistently occurred during adolescence; such trends were more significant among girls. The findings of this study suggest that gender-specific interventions aiming to promote PA and concurrently to limit SED should be implemented for active lifestyles among children and adolescents as early as possible.

## Abbreviations

aOR: adjusted odds ratio
BMI: body mass index
CA: cluster analysis
CI: confidence intervals
LCA: latent cluster analysis
MVPA: moderate to vigorous physical activity
OR: odds ratio
PA: physical activity
SED: sedentary behavior
ST: screen time
uOR: unadjusted odds ratio

## References

[1] Gabriel KKP, Morrow JR, Woolsey AL (2012). Framework for physical activity as a complex and multidimensional behavior. J Phys Act Health.

[2] Poitras VJ, Gray CE, Borghese MM, Carson V, Chaput J-P, Janssen I (2016). Systematic review of the relationships between objectively measured physical activity and health indicators in school-aged children and adolescents. Appl Physiol Nutr Metab.

[3] Carson V, Hunter S, Kuzik N, Gray CE, Poitras VJ, Chaput J-P (2016). Systematic review of sedentary behaviour and health indicators in school-aged children and adolescents: an update. Appl Physiol Nutr Metab.

[4] Biddle SJ, Pearson N, Ross GM, Braithwaite R (2010). Tracking of sedentary behaviours of young people: a systematic review. Prev Med.

[5] World Health Organization. Global recommendations on physical activity for health. Geneva: World Health Organization; 2010. http://www.who.int/ncds/prevention/physical-activity/factsheet_young_people/en/. Accessed 8 Nov 2018.

[6] Tremblay MS, Carson V, Chaput JP, Connor Gorber S, Dinh T, Duggan M (2016). Canadian 24-hour movement guidelines for children and adolescents: an integration of physical activity, sedentary behaviour, and sleep. Appl Physiol Nutr Metab.

[7] Dumith SC, Gigante DP, Domingues MR, Kohl HW (2011). Physical activity change during adolescence: a systematic review and a pooled analysis. Int J Epidemiol.

[8] Cooper AR, Goodman A, Page AS, Sherar LB, Esliger DW, Sluijs EM (2015). Objectively measured physical activity and sedentary time in adolescents: the international children’s accelerometry database (ICAD). Int J Behav Nutr Phys Act.

[9] Hallal PC, Andersen LB, Bull FC, Guthold R, Haskell W, Ekelund U (2012). Global physical activity levels: surveillance progress, pitfalls, and prospects. Lancet.

[10] Tremblay MS, Barnes JD, González SA, Katzmarzyk PT, Onywera VO, Reilly JJ (2016). Global matrix 2.0: report card grades on the physical activity of children and adolescents comparing 38 countries. J Phys Act Health.

[11] Barnes JD, Cameron C, Carson V, Chaput JP, Faulkner GEJ, Janson K (2016). Results from Canada’s 2016 ParticipACTION report card on physical activity for children and adolescents. J Phys Act Health.

[12] Wilkie H, Standage M, Sherar L, Cumming S, Parnell C, Davis A (2016). Results from England’s 2016 report card on physical activity for children and adolescents. J Phys Act Health.

[13] Katzmarzyk PT, Denstel KD, Beals K, Bolling C, Wright C, Crouter SE (2016). Results from the United States of America’s 2016 report card on physical activity for children and adolescents. J Phys Act Health.

[14] Ishii K, Shibata A, Adachi M, Nonoue K, Oka K (2015). Gender and grade differences in objectively measured physical activity and sedentary behavior patterns among Japanese children and adolescents: a cross-sectional study. BMC Public Health.

[15] Song Y, Yang HI, Lee E-Y, Yu M-S, Kang MJ, Kang HJ (2016). Results from South Korea’s 2016 report card on physical activity for children and adolescents. J Phys Act Health.

[16] Tudor-Locke C, Ainsworth BE, Adair LS, Du S, Popkin BM (2003). Physical activity and inactivity in Chinese school-aged adolescents: the China health and nutrition survey. Int J Obes Relat Metab Disord.

[17] Liu Y (2017). Promoting physical activity among Chinese adolescents: no time to wait. J Sport Health Sci.

[18] Li F, Liu Y, Zhu W, Harmer P (2016). China's challenges in promoting physical activity and fitness. Lancet.

[19] Cui Z, Hardy LL, Dibley MJ, Bauman A (2011). Temporal trends and recent correlates in sedentary behaviours in Chinese children. Int J Behav Nutr Phys Act.

[20] Fan X, Cao ZB (2017). Physical activity among Chinese school-aged children: national prevalence estimates from the 2016 physical activity and fitness in China—the adolescents study. J Sport Health Sci.

[21] Cai Y, Zhu X, Wu X (2017). Overweight, obesity, and screen-time viewing among Chinese school-aged children: national prevalence estimates from the 2016 physical activity and fitness in China—the adolescents study. J Sport Health Sci.

[22] Liu Y, Tang Y, Cao ZB, Chen PJ, Zhang JL, Zhu Z, Zhuang J (2016). Results from Shanghai’s (China) 2016 report card on physical activity for children and adolescents. J Phys Act Health.

[23] Duan J, Hu H, Wang G, Arao T (2015). Study on current levels of physical activity and sedentary behavior among middle school students in Beijing. China Plos One.

[24] Ainsworth BE (2016). How physically active are our children? A global view. J Sport Health Sci.

[25] Sallis JF, Prochaska JJ, Taylor WC (2000). A review of correlates of physical activity of children and adolescents. Med Sci Sport Exer.

[26] Bauman AE, Reis RS, Sallis JF, Wells JC, Loos RJF, Martin BW (2012). Correlates of physical activity: why are some people physically active and others not?. Lancet.

[27] Lu C, Stolk RP, Sauer PJJ, Sijtsma A, Wiersma R, Huang G (2017). Factors of physical activity among Chinese children and adolescents: a systematic review. Int J Behav Nutr Phys Act.

[28] Stierlin AS, Lepeleere S, Cardon G, Dargent-Molina P, Hoffmann B, Murphy MH (2015). A systematic review of determinants of sedentary behaviour in youth: a DEDIPAC-study. Int J Behav Nutr Phys Act.

[29] Verloigne M, Loyen A, Hecke L, Lakerveld J, Hendriksen I, Bourdheaudhuij I (2016). Variation in population levels of sedentary time in European children and adolescents according to cross-European studies: a systematic literature review within DEDIPAC. Int J Behav Nutr Phys Act.

[30] Arundell L, Fletcher L, Salmon J, Veitch J, Hinkley T (2016). The correlates of after-school sedentary behavior among children aged 5–18 years: a systematic review. BMC Public Health.

[31] Morgan K, Hallingberg B, Littlecott H, Murphy S, Fletcher A, Roberts C (2016). Predictors of physical activity and sedentary behaviours among 11-16 year olds: multilevel analysis of the 2013 health behaviour in school-aged children (HBSC) study in Wales. BMC Public Health.

[32] Ottevaere C, Huybrechts I, Benser J, Bourdeaudhuij ID, Cuencagarcia M, Dallongeville J (2011). Clustering patterns of physical activity, sedentary and dietary behavior among European adolescents: the HELENA study. BMC Public Health.

[33] Leech RM, McNaughton SA, Timperio A (2014). The clustering of diet, physical activity and sedentary behavior in children and adolescents: a review. Int J Behav Nutr Phys Act.

[34] Roman-Viñas B, Chaput JP, Katzmarzyk PT, Fogelholm M, Lambert EV, Maher C (2016). Proportion of children meeting recommendations for 24-hour movement guidelines and associations with adiposity in a 12-country study. Int J Behav Nutr Phys Act.

[35] Patnode CD, Lytle LA, Erickson DJ, Sirard JR, Barr-Anderson DJ, Story M (2011). Physical activity and sedentary activity patterns among children and adolescents: a latent class analysis approach. J Phys Act Health.

[36] Saunders TJ, Gray CE, Poitras VJ, Chaput JP, Janssen I, Katzmarzyk PT (2016). Combinations of physical activity, sedentary behaviour and sleep: relationships with health indicators in school-aged children and adolescents. Appl Physiol Nutr Metab..

[37] Liu Y, Wang M, Tynjälä J, Lv Y, Villberg J, Zhang Z (2010). Test-retest reliability of selected items of health behaviour in school-aged children (HBSC) survey questionnaire in Beijing. China BMC Med Res Methodol.

[38] Li F, Chen P (2017). Addressing the public health concerns of physical inactivity, low levels of fitness, and unhealthy weight among Chinese school-aged children. J Sport Health Sci.

[39] van der Ploeg HP, Hillsdon M (2017). Is sedentary behaviour just physical inactivity by another name?. Int J Behav Nutr Phys Act.

[40] Yang B, Chen S, Laurson KR, Kim Y, Saintmaurice PF, Welk GJ (2016). The associations of adolescents physical activity and screen time with fatness and fitness: the 2012 NHANES national adolescents fitness survey. PLoS One.

[41] Chen S, Kim Y, Gao Z (2014). The contributing role of physical education in youth’s daily physical activity and sedentary behavior. BMC Public Health.

[42] Hoffmann B, Kettner S, Wirt T, Wartha O, Hermeling L, Steinacker JM (2017). Sedentary time among primary school children in south-West Germany: amounts and correlates. Arch Public Health.

[43] Tremblay MS, LeBlanc AG, Kho ME, Saunders TJ, Larouche R, Colley RC (2011). Systematic review of sedentary behaviour and health indicators in school-aged children and youth. Int J Behav Nutr Phys Act.

[44] Jones RA, Hinkley T, Okely AD, Salmon J (2013). Tracking physical activity and sedentary behavior in childhood: a systematic review. Am J Prev Med.

[45] Telama R, Yang X, Leskinen E, Kankaanpää A, Hirvensalo M, Tammelin T (2014). Tracking of physical activity from early childhood through youth into adulthood. Med Sci Sport Exer.

[46] Anderssen N, Wold B, Torsheim T (2005). Tracking of physical activity in adolescence. Res Q Exerc Sport.

[47] Telama R (2009). Tracking of physical activity from childhood to adulthood: a review. Obes Facts.

[48] Sallis JF (2000). Age-related decline in physical activity: a synthesis of human and animal studies. Med Sci Sport Exer.

[49] Craggs C, Corder K, van Sluijs EM, Griffin SJ (2011). Determinants of change in physical activity in children and adolescents: a systematic review. Am J Prev Med.

[50] Patnode CD, Lytle LA, Erickson DJ, Sirard JR, Barranderson D, Story M (2010). The relative influence of demographic, individual, social, and environmental factors on physical activity among boys and girls. Int J Behav Nutr Phys Act.

[51] Huang WY, Wong SH, Salmon J (2013). Correlates of physical activity and screen-based behaviors in Chinese children. J Sci Med Sport.

[52] Welk GJ, Corbin CB, Dale D (2000). Measurement issues in the assessment of physical activity in children. Research Q Exer Sport.

[53] Sirard JR, Pate RR (2001). Physical activity assessment in children and adolescents. Sports Med.

[54] Kang M, Rowe DA (2015). Issues and challenges in sedentary behavior measurement. Measu in Physi Educa Exer Sci.

[55] Atkin AJ, Trish G, Clemes SA, Thomas Y, Charlotte E, Soren B (2012). Methods of measurement in epidemiology: sedentary behaviour. Int J Epidemiol.

[56] Sallis JF (1991). Self-report measures of children's physical activity. J Sch Health.

